# Evaluation of five CAD/CAM materials by microstructural characterization and mechanical tests: a comparative in vitro study

**DOI:** 10.1186/s12903-017-0458-2

**Published:** 2018-01-08

**Authors:** Nesrin Sonmez, Pinar Gultekin, Volkan Turp, Gokhan Akgungor, Deniz Sen, Eitan Mijiritsky

**Affiliations:** 1 Beykent University Vocational School, Dental Services, Dental Prosthesis Technology, Istanbul, Turkey; 2 Faculty of Dentistry, Department of Prosthodontics, Istanbul University, 34093 Fatih, Istanbul Turkey; 3 Sackler Faculty of Medicine, Department of Otolaryngology Head and Neck and Maxillofacial Surgery, Tel-Aviv Sourasky Medical Center, Tel-Aviv University, Tel Aviv, Israel

**Keywords:** CAD/CAM, Nano-ceramic resin, Ceramic-polymer, Thermocycling

## Abstract

**Background:**

Polymer infiltrated ceramics and nano-ceramic resins are the new restorative materials which have been developed in order to enhance the adverse properties of glass-matrix ceramics and resin composites. The aim of the present in vitro study was to evaluate the characteristics of various CAD/CAM materials through mechanical, microstructural, and SEM analysis.

**Methods:**

Five test groups (*n* = 22) were formed by using the indicated CAD/CAM blocks: VITA Enamic (VITA Zahnfabrik), Lava Ultimate (3 M ESPE), IPS e.max CAD (Ivoclar Vivadent), IPS Empress CAD (Ivoclar Vivadent), and VITA Mark II (VITA Zahnfabrik). Two specimens from each test group were used for XRD and EDS analysis. Remaining samples were divided into two subgroups (*n* = 10). One subgroup specimens were thermocycled (5 °C to 55 °C, 30s, 10,000 cycles) whereas the other were not. All of the specimens were evaluated in terms of flexural strength, Vickers hardness, and fracture toughness. Results were statistically analyzed using two-way ANOVA, one-way ANOVA, Tukey’s HSD, and Student’s t tests (α = .05). Fractured specimens were evaluated using SEM.

**Results:**

The highest Vickers microhardness value was found for VITA Mark II (*p* < .001), however flexural strength and fracture toughness results were lowest conversely (*p* < .05). IPS e.max CAD was found to have the highest flexural strength (p < .001). Fracture toughness of IPS e.max CAD was also higher than other tested block materials (*p* < .001). Lava Ultimate and VITA Enamic’s mechanical properties were affected negatively from thermocycling (*p* < .05). Microhardness, flexural strength, and fracture toughness values of Lava Ultimate and VITA Enamic were found to be similar to VITA Mark II and IPS Empress CAD groups.

**Conclusions:**

It should be realised that simulated aging process seem to affect ceramic-polymer composite materials more significantly than glass ceramics.

## Background

Glass-matrix ceramics and resin composites are frequently used materials for CAD/CAM (Computer Aided Design/Computer Aided Manufacturing) restorations due to enhanced mechanical and optical properties [1, 2]. Although they are well established and successful materials, they suffer from several disadvantages. Glass-matrix ceramics have mechanical problems such as brittleness and abrasion on the opposing dentition due to hardness [3]. Resin composites may undergo wear, missing surface polish and stability of color [2, 4–6]. In order to improve the unfavourable properties of glass-matrix ceramics and resin composites, new restorative materials have been developed which are called polymer infiltrated ceramics and nano-ceramic resins for usage with CAD/CAM systems [7]. VITA Enamic and Lava Ultimate are examples of this class of materials.

VITA Enamic is composed of a ceramic part (75% by volume) and a polymer part (25% by volume). Its ceramic phase includes 23% Al_2_O_3_ and the polymer part contains urethane dimethacrylate (UDMA) and triethylene glycol dimethacrylate (TEGDMA) [6, 7]. It is formed by penetration of presintered ceramic phase into polymer using capillary activity [6]. VITA Enamic was reported to have lower translucency in comparison to Lava Ultimate and glass-matrix ceramics due to relatively high amount of Al_2_O_3_, therefore it is advised to be used for minimally invasive reconstructions, inlays, onlays, and posterior crowns [8–11].

On the other hand, Lava Ultimate Restorative includes nanoceramic particles deep-seated in a highly cross-linked resin network. The combination of discrete silica and zirconia nanoparticles with zirconia-silica nanoclusters reduces the space between the filler particles [6, 7]. The inorganic part containing zirconia and silica nanoparticles forms approximately 80 wt.% of the material (69% SiO_2_, 31% ZrO_2_) whereas the organic polymer part about 20 wt.% contains UDMA (urethane dimethacrylate) and Bis-EMA (bisphenol A polyethethylene glycol diether dimethacrylate) [6, 7, 11]. Lava Ultimate has superior translucency in comparison to VITA Enamic and glass-matrix ceramics with its smaller filler size, therefore it can be used for inlays, onlays, and veneers whereas it is no longer indicated for full crowns due to debonding problems [8–10, 12, 13].

It has been previously reported that the combination of ceramic and polymer phases gives these materials stability, flexural strength, elasticity, and hardness similar to natural tooth structure [14, 15]. The presence of a polymer network helps absorbing the chewing forces more than glass ceramics [6]. Polymer infiltrated ceramics have been reported to have a flexural strength of approximately 150 MPa whereas nano-ceramic resins have a flexural strength of 200 MPa [6, 7, 16, 17]. Their chemical differences due to the composition of the filler and the matrix-filler coupling mechanism may cause varied resistance to the materials’ mechanical and chemical degradation [18, 19].

Polymer infiltrated ceramics and nano-ceramic resins are reported to have several advantages compared to conventional restorative materials, however, available information on the properties of these materials after a prolonged usage period are limited [19, 20]. The purpose of this study was to investigate the chemical contents and to compare mechanical behavior of polymer infiltrated ceramics and nano-ceramic resins under different conditions in comparison with clinically proven ceramic CAD/CAM blocks. The first hypothesis was that polymer infiltrated ceramics and nano-ceramic resins have higher flexural strength compared to glass-matrix ceramic. The second hypothesis was that polymer infiltrated ceramics and nano-ceramic resins have lower Vickers hardness and fracture toughness values compared to glass-matrix ceramics. The third hypothesis was that mechanical properties of nano-ceramic resins and polymer infiltrated ceramics might be more affected by thermocycling compared to glass-matrix ceramics.

## Methods

Five monolithic high translucent CAD/CAM block materials (A2 HT and 2 M2 HT shades) were investigated in the study; a feldspathic ceramic VIT: VITA Mark II (VITA Zahnfabrik, Bad Säckingen, Germany), a leucite based ceramic EMP: IPS Empress CAD (Ivoclar Vivadent, Schaan Liechtenstein), a lithium disilicate ceramic MAX: IPS e.max CAD (Ivoclar Vivadent, Schaan, Liechtenstein), a nanoceramic resin ULT: Lava Ultimate (3 M ESPE, Neus, MN, USA), and a hybrid ceramic ENA: VITA Enamic (VITA Zahnfabrik, Bad Säckingen, Germany). Power analysis using G*Power statistical software (G*Power Ver. 3.0.10, Franz Faul, Universität Kiel, Germany) was performed to determine the sample size. A total of 10 samples per group were set considering Power: 0.80, α:0.05, effect size: 2.4 and SD: 20 for mechanical tests. The codes used for the materials and their classifications are displayed in Table 1.

**Table 1:** The blocks tested in the study

Materials tested	Code	Color-Batch no.	Classification	Manufacturer	Chemical content ^a^ (wt%)	Clinical Indications ^a^
VITA Mark II	VIT	2M2C-16,630	Feldspathic glass-matrix ceramic	VITA Zahnfabrik, Germany	56–64% SiO_2,_ 20–23% Al_2_O_3,_ 6–9% Na_2_O, 6–8% K_2_O	Veneers, inlays, onlays, anterior and posterior crowns.
IPS Empress CAD	EMP	HT A2-N74772	Leucite based glass-matrix ceramic	Ivoclar Vivadent AG, Liechtenstein	64.9% SiO_2_, 16.25% Al_2_O_3_, 11.85% K_2_O, 5.37% Na_2_O, 1.56% CaO	Veneers, inlays, onlays, anterior and posterior crowns.
IPS e.max CAD	MAX	HT A2-L02944	Lithium disilicate based glass-matrix ceramic	Ivoclar Vivadent AG, Liechtenstein	58–80% SiO_2_, 11–19% Li_2_O, 0–13% K_2_O, 0–8% ZrO_2_ 0–5% Al_2_O_3,_	Veneers, inlays, onlays, anterior and posterior crowns, anterior and posterior implant abutments, three-unit bridges up to premolars.
Lava Ultimate	ULT	A2 HT-N420014	Resin Nanoceramic	3 M ESPE, USA	80% inorganic (69% SiO_2_, 31% ZrO_2_) 20% organic	Veneers, inlays, onlays.
Vita Enamic	ENA	2 M2 HT-59620	Hybrid ceramic	VITA Zahnfabrik, Germany	86% inorganic (58–63% SiO_2_, 20–23% Al_2_O_3_, 9–11% Na_2_O, 4–6% K_2_O, 0.1% ZrO_2_) 14% organic	Veneers, inlays, onlays, anterior and posterior crowns.

^a^As disclosed by manufacturers

### Preparation of test specimens

Test specimens were fabricated using stainless steel bars (1.2x4x14 mm) which would be used as guides. Cercon CAD/CAM system (Cercon Degudent, Dentsply, NY, USA) was used for the scanning of the stainless steel bar guides and copy milling of the ceramic test specimens. Twenty-two samples were prepared from each block material. Following the milling, all specimens were consecutively polished with 600, 800 and 1000 grit Silicon Carbide (SiC) papers (Struers, Copenhagen, Denmark) with water in a grinding device (Struers Labo-pol 5, Struers, Copenhagen, Denmark) to the final dimensions of 1.2 ± 0.2 × 4 ± 0.2 × 14 ± 0.2 mm. The dimensions of specimens were checked with a digital caliper (Humboldt, China).

### Micromorphology analysis

The two intact specimens from each group were separated randomly to be used in micromorphology analysis. One specimen was used for X-ray diffraction (XRD) and the other for energy dispersive X-ray spectroscopy analysis (EDS).

XRD was carried out to determine the crystalline phases in the materials studied. The specimens were scanned by an X-ray diffractometer (Rigaku Miniflex, Texas, USA) using Cu-Kα radiation from 10° to 90° 2θ degrees with 0.04°step size and 5-step intervals.

EDS analysis was carried out to investigate the chemical content of the materials used in the study. All specimens were sputter-coated using carbon after being air dried. Surface examination of the specimens was made with a scanning electron microscope (JSM 7000F, JEOL, Japan). Each determined area was analysed under 5.00 kV acceleration voltage for duration of 100 s.

### Thermocycling

Each group containing 20 samples were randomly divided into two subgroups (*n* = 10). First subgroup specimens (n = 10) were stored in dry environment whereas the second subgroup (n = 10) was aged with 10.000 thermal cycles (5 °C to 55 °C, dwelling time 30 s) with the aid of a thermocycling machine (Salubris-technica, Dentester, Istanbul, Turkey).

### Flexural strength test

Following the thermocycling, the flexural strength of all the samples were investigated using the three point bending test conducted on a transversal testing machine (Shimadzu AG-IS, Shimadzu, Kyoto, Japan) with 12 mm support span [21]. The bars were loaded until fracture with a crosshead speed of 0.5 mm/min. The flexural strength was calculated by the formula as follows:

$$ \boldsymbol{\sigma} \boldsymbol{f}=\frac{\mathbf{3}\boldsymbol{Fl}}{\mathbf{2}\boldsymbol{w}{\boldsymbol{h}}^{\mathbf{2}}} $$

Where σf is the flexural strength, F is the fracture load, l is the roller span, w the width and h the height of the bar.

### Vickers hardness test

All of the specimens which were fractured in the flexural strength test were evaluated for the Vickers hardness measurement. Hardness measurements were made on selected points far from the fracture line using the digital camera of the tester. Vickers hardness measurements were performed with a hardness tester (UHL VMHT, Walter Uhl, Asslar, Germany) using a 200 gf load for 10 s dwell time. Vickers Hardness value was calculated with the following formula;

$$ \boldsymbol{HV}=\boldsymbol{1.8544}\ \left(\boldsymbol{F}/{\boldsymbol{d}}^{\boldsymbol{2}}\right) $$

Where HV is the Vickers hardness number, F is the load and d is the indentation diagonal length.

### Fracture toughness evaluation

For the evaluation of the samples’ fracture toughnesses, following the microhardness test, the Vickers indentations were observed under optical microscope. Crack lengths were obtained and fracture toughness was calculated by the formula as follows:

$$ {\mathbf{K}}_{\mathbf{IC}}=\mathbf{0.203}{\left(\mathbf{c}/\mathbf{a}\right)}^{-\mathbf{3}/\mathbf{2}}{\mathbf{Ha}}^{\mathbf{1}/\mathbf{2}} $$

Where K_IC_ is the fracture toughness, c is the half-diagonal of the indentation, a is the average median/radial crack length, and H is Vickers hardness number.

### Scanning electron microscopy (SEM) analysis

One fractured specimen per group was selected randomly and gold coated for scanning electron microscope (SEM) observation. Scanning electron microscopy (Rigaku Miniflex, TX, USA) was employed to examine the fraction areas. The embedded specimens were analyzed by secondary electron detector at 5.00 kV.

### Statistical analysis

Statistical analysis was carried out with a specific software (IBM SPSS Statistics for Windows Version 22.0; IBM Corp., NY, USA). Normalities of distributions were explored by means of the Kolmogorov–Smirnov test and the groups were found to be distributed normally. The effect of the material and aging independent variables on the flexural strength, Vickers hardness, and fracture toughness dependent variables was evaluated with two-way analysis of variance (ANOVA). Repeated measures analysis was performed with one-way ANOVA for subgroups (*n* = 10). Tukey’s post hoc comparison was used to identify which groups differed from one another. Student’s t-test was performed to determine the significance of the differences between two groups before and after thermocycling. *P* < .05 was considered significant.

Flexural strength data variability was evaluated using a two-parameter Weibull cumulative distribution function. Weibull cumulative distribution was calculated using the following equation:

$$ \mathbf{P}\left(\boldsymbol{\upsigma} \right)=\mathbf{1}-\mathbf{\exp}\left[-\left(\boldsymbol{\upsigma} /\boldsymbol{\upsigma} \mathbf{0}\right)\mathbf{m}\right] $$

Where P(σ) is the probability of failure, σ is the fracture stress, σ0 is the characteristic parameter corresponding to the fracture probability of 63.2%, and m is the Weibull modulus. Weibull distribution graphs were obtained using Weibull-Ease 16.0 software (Applications Research, Inc. Golden Valley, MN, USA).

Weibull modulus was calculated by constructing a plot with lnln [(1/1 − P(σ))] on the ordinate and a corresponding lnσ on the abscissa and calculating the slope of the fitted line, where slope equals m; the Weibull modulus.

## Results

### Micromorphology analysis

Results of EDS analysis were summarized in Table 2. According to the EDS analysis ULT includes two types of components: resin matrix and ceramic filler structure. ENA has polymer and ceramic network. ULT and ENA include equal ratios of ceramic (inorganic) components. However ULT includes ZrO_2_, ENA has Al_2_O_3_, Na_2_O and K_2_O in its inorganic network. Two materials have similar ratios of SiO_2_. VIT, EMP, and MAX displayed high ratios of SiO_2_, Al_2_O_3_ and other metal oxides.

**Table 2:** Chemical content of the tested materials determined with EDS analysis

Materials tested	Chemical content according to EDS analysis
LAVA Ultimate	82.1% inorganic (69% SiO_2_, 31%ZrO_2_), 17.9% organic
Vita Enamic	83.2% inorganic (64.2% SiO_2_, 20.6% Al_2_O_3_, 8.6% Na_2_O, 6.5%K_2_O), 16.8% organic
VITA Mark II	64% SiO_2_, 20% Al_2_O_3_, 9% Na_2_O, 6% K_2_O
IPS Empress CAD	64.9% SiO_2_, 16.25% Al_2_O_3_, 11.85% K_2_O, 5.37% Na_2_O, 1.56% CaO
IPS e.max CAD	80.1% SiO_2_, 11.9% Li_2_O, 4.8% P_2_O_5_, 4.4% K_2_O, 4.4% Al_2_O_3_

**Fig. 1:**
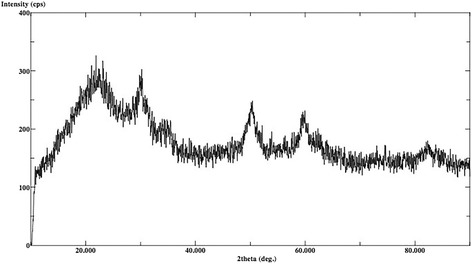
X-ray diffraction patterns of Lava Ultimate

**Fig. 2:**
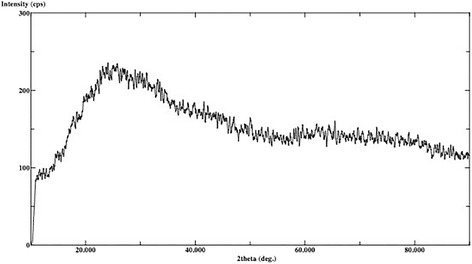
X-ray diffraction patterns of Vita Enamic

**Fig. 3:**
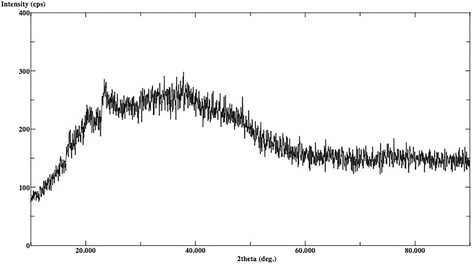
X-ray diffraction patterns of VITA Mark II

**Fig. 4:**
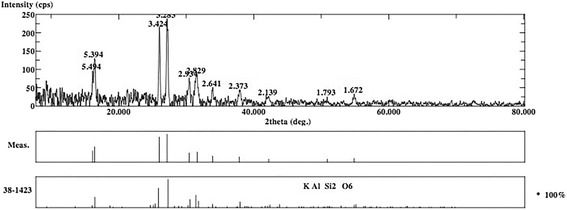
X-ray diffraction patterns of IPS Empress CAD

**Fig. 5:**
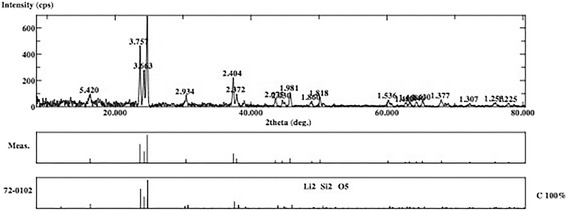
X-ray diffraction patterns of IPS e.max CAD

The XRD analysis displayed ULT, ENA and VIT as amorphous materials without different phases (Figs. 1, 2 and 3). There are no dominant peaks deflected from the surfaces of these materials. XRD also displayed (Figs. 4 and 5) IPS Empress CAD and IPS e.max CAD had randomly oriented crystallization focus points. Dominant peaks refer to leucite (KAlSi_2_O_6_) crystals for IPS Empress CAD and lithium disilicate crystals (Li_2_Si_2_O_5_) for IPS e.max CAD.

### Flexural strength

**Fig. 6:**
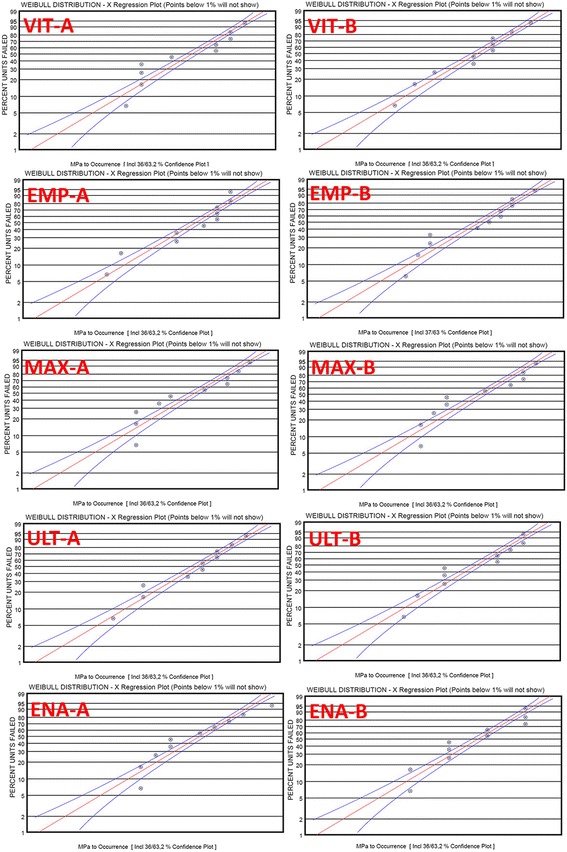
Weibull distribution graphs of groups. VIT A is non-aged, VIT B is aged group. EMP A is non-aged, EMP B is aged group. MAX A is non-aged, MAX B is aged group. ULT A is non-aged, ULT B is aged group. ENA A is non-aged, ENA B is aged group

Two-way ANOVA showed that the type of tested material and aging, and the interaction between these parameters were significant (*p* < .01) (Table 3). The results of the one-way ANOVA revealed that the differences between the flexural strength of the materials were statistically significant (*p* < .01) (Table 4). MAX group had significantly higher flexural strength compared to other groups whereas VIT and EMP had the lowest values (*p* < .01). According to the *Student’s* t-test, flexural strengths of ceramic-polymer composite materials (ULT and ENA) were significantly decreased after thermocycling (*p* < .01). No significant decrease in flexural strength of glass ceramics (MAX, EMP, and VIT) was detected after thermocycling (*p* > .05). Weibull distribution of groups are displayed in Fig. 6 and Weibull moduli of groups are shown in Table 3 ranging from 12.1 to 14.6.

**Table 3:** Results of two-way ANOVA for flexural strength, Vickers hardness, and fracture toughness

Test method	Source of variation	Sum of squares	df	Mean square	F	*p*
Flexural strength	Material	820,499.7	4	205,124.9	20,258.7	0.001**
	Aging	8716.5	1	8716.5	860.9	0.001**
	Material*Aging	12,610.5	4	3152.6	311.4	0.001**
	Error	911.3	90	10.1		
	Total	4,101,565.2	100			
Vickers hardness	Material	526.6	4	131.6	156,616.9	0.001**
	Aging	0.4	1	0.4	491.9	0.001**
	Material*Aging	0.8	4	0.2	246.2	0.001**
	Error	0.1	90	0.0		
	Total	2344.4475	100			
Fracture toughness	Material	15.2	4	3.8	3984.7	0.001**
	Aging	0.8	1	0.8	851.4	0.001**
	Material*Aging	1.2	4	0.3	311.6	0.001**
	Error	0.1	90	0.0		
	Total	278.2	100			

Two-way ANOVA

**p < 0.01

**Table 4:** The results of flexural strength test Weibull moduli. Different capital letters in the same column and different small letters in the same row indicate significant difference (*p* < .01)

	Flexural Strength (MPa)		^*1*^ *p*	Weibull Modulus (M)	
	Before thermocycling	After thermocycling		Before thermocycling	After thermocycling
	Mean ± Sd	Mean ± Sd			
VIT	112.4 ± 3.2 D,a	112.1 ± 2.3 B,a	0.853	13.2	13.3
EMP	134.5 ± 3.3 C,D,a	134.7 ± 3.8 B,a	0.908	14.6	14.3
MAX	359.2 ± 4.2 A,a	357.7 ± 3.7 A,a	0.406	12.1	13.1
ULT	191.2 ± 2.7 B,a	140.4 ± 3.5 B,b	0.001**	13.7	12.9
ENA	152.1 ± 2.9 C,a	111.1 ± 1.2 B,b	0.001**	14.2	13.6
^*2*^ *p*	0.001**	0.001**			

**p < 0.01

^1^Student’s t test

^2^One-way ANOVA test

### Vickers hardness

Two-way ANOVA showed that the type of tested material and aging, and the interaction between these parameters were significant (*p* < .01) (Table 3). The results of one-way ANOVA revealed that the differences between the Vickers hardness of the materials were statistically significant (*p* < .01) (Table 5). The microhardness values of VIT, EMP, and MAX groups were significantly higher compared to ULT and ENA groups (*p* < .01). There were no statistically significant difference between microhardness values of VIT, EMP, and MAX groups (*p* > .05). There were also no statistically difference between the microhardness values of ULT and ENA groups (*p* > .05). According to the *Student’s* t test, Vickers hardness of ceramic-polymer composite materials (ULT and ENA) were significantly decreased after thermocycling (*p* < .01). There was no significant decrease in Vickers hardness of glass ceramics (MAX, EMP, and VIT) after thermocycling (*p* > .05).

**Table 5:** Vickers hardness of materials before and after thermocycling. Different capital letters in the same column and different small letters in the same row indicate significant difference (*p* < .01)

Vickers Hardness (VHN)			^1^ *p*
	Before Thermocycling	After Thermocycling	
	Mean ± SD	Mean ± SD	
VIT	6.4 ± 0.1 A,a	6.3 ± 0.1 A,a	0.733
EMP	6.1 ± 0.1 A,a	6.1 ± 0.1 A,a	0.196
MAX	5.8 ± 0.1 A,a	5.8 ± 0.1 A,a	0.946
ULT	1.1 ± 0.1 B,a	0.8 ± 0.1 B,b	0.001**
ENA	2.3 ± 0.1 B,a	1.9 ± 0.1 B,b	0.001**
^2^ *p*	0.001**	0.001**	

**p < 0.01

^1^Student’s t test

^2^One-way ANOVA Test

### Fracture toughness

Two-way ANOVA showed that the type of tested material and aging, and the interaction between these parameters were significant (*p* < .01) (Table 3). The results of the one-way ANOVA revealed that the differences between the fracture toughness of the materials were statistically significant (*p* < .01) (Table 6). The fracture toughness of VIT, EMP, and MAX groups were significantly higher compared to ULT and ENA groups (*p* < .01). There were no statistically significant difference between fracture toughness of VIT, EMP, and MAX groups (*p* > .05). There were also no statistically difference between fracture toughness of ULT and ENA groups (*p* > .05). According to the Student’s t test, fracture toughness of ceramic-polymer composite materials (ULT and ENA) were significantly decreased after thermocycling (*p* < .01). There was no significant decrease in fracture toughness of glass ceramics (MAX, EMP, and VIT) after thermocycling (*p* > .05).

**Table 6:** The results of fracture toughness test and statistical analysis

Fracture Toughness (MPa•m^1/2^)			^1^ *p*
	Before thermocycling	After thermocycling	
	Mean ± Sd	Mean ± Sd	
VIT	2.34 ± 0.04 A,a	2.33 ± 0.03 A,a	0.853
EMP	1.90 ± 0.03 A,a	1.88 ± 0.03 A,a	0.406
MAX	1.67 ± 0.03 A,a	1.63 ± 0.03 A,a	0.908
ULT	1.29 ± 0.03 B,a	1.10 ± 0.04 B,b	0.001**
ENA	1.23 ± 0.02 B,a	1.02 ± 0.01 B,b	0.001**
^2^ *p*	0.001**	0.001**	

**p < 0.01

^1^Student’s t test

^2^One-way ANOVA test

### SEM micrographs of fractured surfaces

**Fig. 7:**
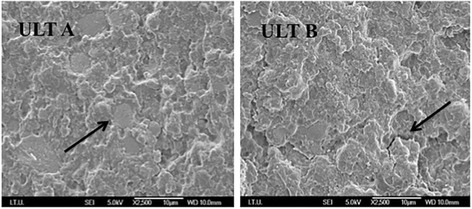
ULT A is the SEM image of non-aged ULT specimen. The black arrow shows inorganic network of the material. ULT B is the SEM image of aged ULT specimen. The black arrow shows the microcracks of the materials

**Fig. 8:**
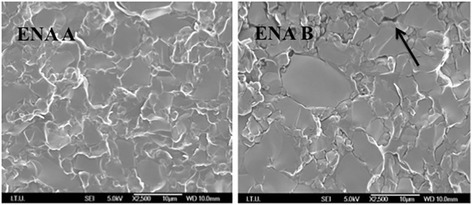
ENA A shows the uniform structure of ENA before thermocycling. ENA B shows the defects of the aged material. The black arrow shows one of many microcracks

**Fig. 9:**
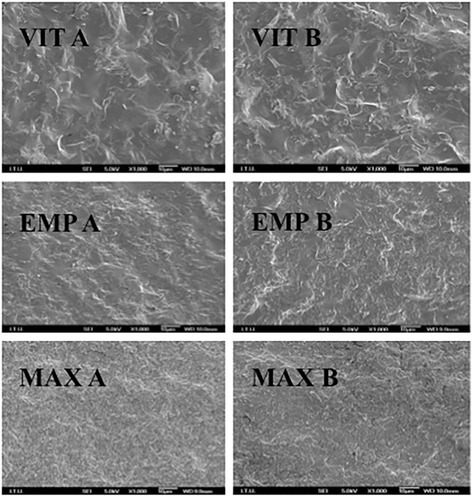
VIT A is non-aged, VIT B is aged VIT specimen. EMP A is non-aged, EMP B is aged EMP specimen. MAX A is non-aged, MAX B is aged MAX specimen

In the SEM observations, ULT displayed microcracks between the inorganic and organic components in the aged specimen (Fig. 7). ENA images displayed defects in the uniform structure of the material after thermocycling (Fig. 8). No distortion of structure were observed after thermocycling in VIT, EMP, and MAX groups (Fig. 9).

## Discussion

The primary hypothesis of the study was rejected; one of the glass-matrix ceramics (MAX) had higher flexural strength compared to polymer infiltrated ceramics and nano-ceramic resins. The secondary hypothesis was accepted; ceramic-polymer composite materials were found to have reduced Vickers hardness and fracture toughness mean values than the glass-matrix ceramics. The tertiary hypothesis of the study was confirmed; ceramic-polymer composite materials were affected by thermocycling.

The present study showed that ceramic-resin composite materials are not superior to all types of glass-matrix ceramics in terms of flexural strength. The reason for the MAX group having the highest flexural strength may be the crystal structure of the material. Wang et al. reported that the mechanical properties of dental glass ceramics are closely related to the crystal structure of the material [22]. XRD analysis of the present study made on MAX specimens demonstrated that lithium disilicate crystals were distributed regularly in the structure of the material (Fig. 5). Lithium disilicate’s crystalline phase could explain the higher flexural strength of MAX compared to ceramic-polymer composite materials [18]. Stawarczyk et al. stated that nanoceramic resins have higher flexural strength than polymer-infiltrated ceramic and leucite based ceramic, but lower than lithium disilicate ceramic in accordance with the present study [10]. A recent study also found that lithium disilicate ceramic has significantly higher flexural strength than nanoceramic, polymer infiltrated ceramic, and feldspathic ceramic groups [9]. The difference was also significant respectively between nanoceramic, polymer-infiltrated ceramic, and feldspathic ceramic groups supporting our results [9]. One study reported that, organic content absorbs the chewing forces and increases the flexural strength of the materials [23]. According to the EDS analysis results, ULT and ENA have organic contents and this might be the reason for higher flexural strength of these materials compared to VIT and EMP (Table 2). Coldea et al. reported in two different studies that ceramic-polymer composite materials have higher flexural strength than glass ceramics due to their organic content [7, 16]. In the present study, the lowest flexural strength values were obtained in VIT group following EMP group (Table 4) which are more brittle than the ceramic-polymer composites, which may suggest a toughening mechanism created by the resin matrix in the microstructure of ULT and ENA [24].

In the present study, ULT has significantly higher flexural strength than ENA (*p* < .001) (Table 4). Although both of these materials contained organic network, the flexural strength of ULT was statistically higher than ENA in accordance with other study results [2, 6, 9]. There are differences in inorganic content of these two materials: ULT has 31% ZrO_2_ and ENA has 20.6% Al_2_O_3_ in the inorganic structure (Table 2). This might be one of the factors contributed the higher flexural strength of ULT than ENA. Another possible explanation for significantly different flexural strength of ULT and ENA may be the differences in composition of the resin matrix, dimension, and dispertion of the filler particles [9]. However, Albero et al. found this difference insignificant which may be due to different test methods and specimen dimensions [25].

Weibull modulus identifies the strength variability, give information on material’s structural homogeneity and has been reported to range from 5 to 15 for dental ceramics [26]. The Weibull moduli of the materials investigated in this study ranged from 12.1 to 14.6 with slight differences among themselves indicating materials’ high structural homogeneity and low variability in strength [18, 27, 28].

Surface hardness is described as relative measure of resistance to permanent surface indentation. Indentation hardness is defined as a factor that affects the capability of getting finished and polished and also resistance of a material to occlusal wear [15, 29]. Fracture toughness can be described as the material’s relative resistance to crack propagation [29]. The present study showed that ceramic-resin composite materials have lower hardness and fracture toughness compared with glass-matrix ceramics. Al-Harbi et al. reported that ULT had lower surface hardness and fracture toughness than VIT [30]. Albero et al. also stated that, ENA and ULT had significantly lower Vickers hardness than MAX, VIT, and EMP in accordance with our results [25]. However, they found that EMP had higher Vickers hardness than VIT, which differs from our study results (Table 5) [25]. The difference may be resulting from variations in test methods. The reason for the ceramic-resin composite materials’ lower hardness and fracture toughness compared to glass ceramics might be due to their lower inorganic content [25]. Previously researchers tried to correlate the hardness with wear resistance [31]. Mörmann et al. reported that ceramic-polymer composite materials show higher excessive material wear than glass-matrix ceramics because of their lower hardness values [32]. Lebon et al. measured tool wear against ENA, ULT, and VIT during milling and reported that milling VIT caused more bur damage than ENA and ULT [33]. It was also reported that less opposing enamel wear occurred against ULT and ENA than MAX [32]. Therefore, it can also be speculated that low hardness of ceramic-polymer composite materials may be an advantage for the protection of opposing teeth from massive wear [7, 14]. However, no clinical follow-up studies exist about these new ceramic-resin composite materials. Future in-vivo and in-vitro studies are required in order to reveal the long-term performance of these materials.

Based on the results of the present study, thermocycling significantly affected the flexural strength, Vickers hardness, and fracture toughness of ULT and ENA but not those of EMP, VIT, and MAX (Tables 4, 5 and 6). Thermocycling may be causing water assimilation in the resin structure, resulting in enlargement of the network of ULT and ENA and simplification in the frictional forces of polymer chains [14, 34, 35]. Moreover, it was speculated that the assimilated water would lead to hydrolysis of the interfacial silane coupling agent providing the chemical bond between the resin matrix and the fillers [29]. Consequently, the flexural strength of nano-ceramic resin (ULT) containing zirconia and aluminum oxide containing polymer infiltrated ceramic (ENA) decreased after thermocycling. However, ceramic based MAX, EMP, and VIT did not show any water absorption. SEM observation of these materials before and after thermocycling supported these results (Fig. 8). The images of homogeneous structures were deteriorated after thermocycling and some microcracks were observed for ULT and ENA (Figs. 6 and 7). On the other hand, no noticeable differences of SEM images were observed before and after thermocycling for MAX, EMP and VIT (Fig. 8).

Present study results are in agreement with results of Thornton who stated that, ULT and ENA were affected by thermocycling but MAX was not affected significantly [36]. In a similar manner, another study reported that flexural strength of VIT showed insignificant reduction after thermocycling due to presence of leucite crystals which may help to stop possible crack propagation initiated by thermal stresses [37]. In two recent studies flexural strength of Lava Ultimate significantly decreased after artificial aging [18, 30], whereas one of them revealed that aging had no effect on Vita Enamic [18]. Lauvauthanon et al. also reported similarly that, Vickers hardness and flexural strength of ULT decreased significantly after thermocycling whereas ENA was not affected significantly due to the differences of filler content [6]. The authors stated that, ENA has %86.4 and ULT has %73.1 filler content and as a consequence of higher filler content, ENA shows lower water absorption than ULT [6]. In contrast, according to EDS analysis in the present study, ENA and ULT were found to have similar ratios of inorganic content (Table 2). Significant decrease in flexural strength of ENA and ULT after thermocycling may be due to similar ratios of inorganic content confirmed by EDS analyis. To the best of authors’ knowledge there is a lack of data regarding the effects of thermocycling comparing glass-matrix ceramics with ceramic-polymer composites for current CAD/CAM materials. Therefore present study may be beneficial as a reference for further research.

One of the limitations of this study may be that applied aging method might be considered short-coming for imitating real clinical conditions. Thermocycling is only one method for investigating the aging of the materials and the materials’ properties may differ following different in vitro aging protocols [18]. Further studies are recommended for simulating clinical conditions more realistic. Another limitation of this study might be the use of one shade (A2 or 2 M2) and translucency (HT) for tested materials. Tested block materials are industrially prefabricated in various shades and may have different translucency properties ranging from low to high. However the effect of the difference in shade and translucency and their relation to the mechanical properties of the materials were not evaluated and further studies on this issue are recommended. The other limitation might be small sample size (less than 30) of tested materials for having adequate Weibull parameters. Nevertheless, several studies exist with lower number of samples for evaluating the structural reliability with Weibull analysis [10, 16, 18]. It would be emphasized that, present study results should be commented cautiously since Weibull analysis was performed for only 10 samples per each group.

Within the limitations of this study, it can be stated that ENA and ULT, also classified as ceramic-polymer composite materials, have significant differences in terms of flexural strength, hardness and fracture toughness when compared to VIT, EMP, and MAX. Clinicians should consider these mechanical properties when deciding on the treatment plan of various clinical situations. It should also be remembered that flexural strength, hardness, and fracture toughness of ENA and ULT are affected negatively by thermocycling and studies investigating their long term success are scarce. Considering that the thermal changes and water absorption are not the only factors that age dental restorative materials, further studies are recommended which would investigate these restorative materials with various aging processes in order to simulate the clinical situation.

## Conclusion

Within the limitations of the study it can be concluded that; flexural strength, Vickers hardness, and fracture toughness of the evaluated materials have significant differences in addition thermocycling affects the aforementioned properties of ULT and ENA negatively. Clinicians are suggested to take these differences into consideration when planning prosthodontic rehabilitation using these materials.

## Abbreviations

ANOVA: Analysis of variance
Bis-EMA: Bisphenol A-polyethethylene glycol diether dimethacrylate
Bis-GMA: Bisphenol A-glycidyl methacrylate
CAD/CAM: Computer aided design/Computer aided manufacturing
EDS: Energy dispersive X-ray spectroscopy
EMP: IPS empress CAD
ENA: VITA enamic
MAX: IPS e.max CAD
SD: Standard deviation
SEM: Scanning electron microscope
SiC: Silicon carbide
TEGDMA: Triethylene glycol dimethacrylate
UDMA: Urethane dimethacrylate
ULT: Lava ultimate
VHN: Vickers hardness
VIT: VITA mark II
XRD: X-ray diffraction
